# Site-Specific MicroRNA Expression May Lead to Different Subtypes in Ulcerative Colitis

**DOI:** 10.1371/journal.pone.0142869

**Published:** 2015-11-16

**Authors:** Raju Ranjha, Surbhi Aggarwal, Sawan Bopanna, Vineet Ahuja, Jaishree Paul

**Affiliations:** 1 School of Life Sciences, Jawaharlal Nehru University, New Delhi, India; 2 Department of Gastroenterology, All India Institute of Medical Sciences, New Delhi, India; SAINT LOUIS UNIVERSITY, UNITED STATES

## Abstract

**Background and Aim:**

Ulcerative Colitis (UC) is a type of inflammatory bowel disease, considered as an important disease of gastrointestinal tract having a huge impact on the health of the patient. Prolonged inflammation of colon in UC patients increases the risk of developing colorectal cancer. MiRNA are reported as a connecting link between inflammation and cancer. Differential miRNA expression is reported in Crohn’s disease (CD) patients involving various regions of the gastrointestinal tract. The current study was performed to dissect out the site specific miRNA expression in the colon biopsy samples of UC patients from Northern India.

**Methods:**

Biopsy samples were collected from UC patients and healthy controls from Rectosigmoid Area (RS) and Ascending Colon (AC). MiRNA expression was compared between patients with RS and AC using a microarray platform. Differential expression was further validated by Real Time PCR analysis. Demographic and pathological data of UC -associated CRC patients was collected from the hospital database and analyzed for assessing the site of cancer.

**Results:**

Upon analysis of data generated on a microarray platform and qRT PCR revealed that the expression of six miRNAs hsa-miR-146b-5p, hsa-miR-335-3p, hsa-miR-342-3p, hsa-miR-644b-3p, hsa-miR-491-3p, hsa-miR-4732-3p were downregulated in patients where RS was involved as compared to AC. The expression of hsa-miR-141-3p was upregulated in patients where RS region was involved as compared to AC. Analysis of the registered UC patient’s database from the hospital revealed that the site of CRC was predomimnantly the rectosigmoid region of the colon in most of the cases.

**Conclusion:**

This is the first study to show the differential expression of miRNA involving different sites of colon in UC patients. Taking our data and previous reports into consideration, we propose that differential miRNA expression during UC perhaps contribute in the development of UC-associated CRC at the rectosigmoid area.

## Introduction

Inflammatory bowel disease (IBD) is a chronic inflammatory disease characterized by severe inflammation of the small bowel and/or the colon leading to recurrent diarrhea and abdominal pain. Crohn`s Disease (CD) and Ulcerative Colitis are two major forms of IBD [1]. Chronic inflammation has been suggested to increase the risk of developing cancer [2]. Patients with ulcerative colitis and Crohn's disease are at increased risk of developing colorectal cancer (CRC) [3–6] Site of UC associated colorectal cancer in patients has been identified as the rectosigmoid area in 59% of cases [4]. It is suggested that periodic colonoscopic surveillance in UC patients is important to identify patients with high risk of colorectal cancer.

MicroRNAs (miRNA) are small endogenous RNA molecules approximately 22 nucleotides long. miRNA can interact with mRNA by binding to 3`UTR and can regulate gene expression at post transcriptional level. It can regulate gene expression either by translational repression or mRNA degradation [7–8]. Each miRNA can target hundreds of mRNA and one mRNA can be targeted my multiple miRNAs [9]. MicroRNAs expression is altered in various inflammatory diseases and cancers including inflammatory bowel disease and CRC [2].

MicroRNAs are reported as a link between chronic inflammation and cancer in various inflammatory diseases [2, 6]. Currently it has been observed that in most of the diseases, changes in the expression of miRNAs are specific to the tissue involved [2]. Differential gene expression is reported in various parts of gastrointestinal tract in mice [10]. Site specific miRNA expression is reported in colon of CD patients [11]. On the basis of available literature, here we proposed to look at site specific miRNA expression in the colon of UC patients. Further, we analyzed the differential expression pattern of the miRNA in order to assess if this dysregulated expression can be linked with the increased incidence of UC- associated CRC at rectosigmoidal area.

Biopsy samples from UC patients from ascending colon and from recto-sigmoid area were collected to evaluate the differential miRNA expression. Microarray analysis was done for evaluating miRNA expression and validated the results using qRT PCR. We found altered expression of some important miRNA which are already reported in various inflammatory diseases and cancer and few which are not reported so far. A retrospective study was performed on the patient’s history from the hospital registry of UC-associated CRC patients for analyzing the site of carcinoma. Our data shows that differences observed in specific miRNA expression could be one of the factors leading to increased risk of CRC at rectosigmoidal area.

## Materials and Methods

### Patients and Tissue Samples

This study included 30 UC patients and 20 non-IBD controls. Colonic pinch biopsies from the ascending colon (AC) and rectosigmoid (RS) area of the colon were collected from UC and control individuals. Out of 30 UC patients, 20 colonic pinch biopsy samples were collected from both ascending colon and Rectosigmoid area of Pancolitis patients. Ten biopsy samples from patients were collected only from rectosigmoid area of Left sided colitis patients. Demographic features of participants are given in Table 1. In case of control individuals, tissue samples were collected from ascending colon of 10 participants and from the rectosigmoid colon of 10 participants. All controls were age and sex matched with Patients. Controls were patients without any symptoms of IBD and without any inflammation of colon. All the biopsy samples were collected in RNA later solution. Diagnosis of the patients was based on clinical findings based on ECCO guidelines [12]. The diagnosis of UC was confirmed by histopathological analysis. This study was approved by the ethical committee of All India Institute of Medical Sciences, New Delhi, India (Ref no. IEC/NP-320/2012) and Institutional Ethics Review Board, JNU (REF no. 2012/student/28). A written consent was taken from all the subjects as well as from the guardian of minor subjects included in this study.

**Table 1:** Demographic features of Study subjects.

	Non IBD control	UC
**No. of Patients**	**20**	**30**
**Sex (M/F)**	**13/7**	**17/13**
**Age (Yr), mean ± SD (Range)**	**34.23 ± 13.1(20–65)**	**34.84 ± 13.75 (17–68)**
**Duration of Disease (Yr), mean ± SD (Range)**	**NA**	**5.89 ± 5.4 (0.5–24)**
**Medication**		
**5ASA**	**0**	**22 (73%)**
**AZT**	**0**	**9 (30%)**
**SSZ**	**0**	**2 (6.66%)**
**Steroids**	**0**	**0**

Yr- Years, SD- Standard Deviation, M-Male, F- Female

### Data collection for UC-associated CRC patients

We collected details of UC associated CRC patients from the patient’s registry maintained by the Gastroenterology department of All India Institute of Medical Sciences (AIIMS), New Delhi, India. Demographic and pathobiological features were analyzed to assess the site of CRC in patients who are suffering from UC.

### Total RNA isolation and RNA quality check

Total RNA was isolated using mirVana miRNA isolation kit (Ambion INC, TX 78744, USA). RNA was stored at -80°C until further analysis. Quality of RNA was analysed by running on 1.5% agarose gel for three bands of 28SrRNA, 18SrRNA and 5SrRNA. Bioanalyser (Agilent Techonologies, California, USA) was used to calculate the RIN value for RNA. Samples with RIN value more than 7 were included for microarray analysis.

### MiRNA Microarray

MiRNA microarray was performed by Exiqon A/S, Denmark. Samples were divided in four categories: UC patients Ascending colon and rectosigmoid area and biopsy samples from controlsfrom the corresponding area. Five RNA samples were pooled in each category in equal concentration [13]. RNA samples were submitted to Exiquon (Denmark) for miRNA expression analysis via miRCURY LNA^™^ microRNA Array (7th gen). The array data has been submitted in NCBI Gene Expression Omnibus (GEO) under the entry series GSE66932. 1.7 fold changes were used as cut off for upregulation and 0.6 fold for downregulation of miRNA expression change.

### Reverse Transcription and quantitative Real Time PCR

Reverse transcription of miRNA was done using gene specific looped primers [14] by revert aid cDNA synthesis kit (Fermentas St. Leon Rot, Germany). Sequence of primer used for reverse transcription and Real time PCR are given in Table 2. Real time PCR was performed using ABI PRISM 7500 Real time PCR system (Applied Biosystems). U6 snRNA was used as internal control. Parameters for real time were—initial denaturation—94°c for 2 min, denaturation- 94°c for 30 sec, annealing 60°c for 1 min for 40 cycles. 20 μl reaction containing 7 μl MQ water, 10 μl SYBR^R^ Green universal PCR Master Mix (Applied Biosystems, California, USA), 1 μl of each forward and reverse primer (4 picomole/μl), 1 ul cDNA. Relative expression of a miRNAs was calculated using Ct values.

**Table 2:** Sequence of primers used for Reverse Transcription and qRTPCR.*

Name		Primers (5′-3′)
**Reverse Universal Primer**		GTGCAGGGTCCGAGGT
**hsa-miR-141-3p**	**RT Primer**	GTCGTATCCAGTGCAGGGTCCGAGGTATTCGCACTGGATACGCCATCTTT
	**Forward**	TAACACTGTCTGGTAAAGAT
**hsa-miR-146b-3p**	**RT Primer**	GTCGTATCCAGTGCAGGGTCCGAGGTATTCGCACTGGATACGAGCCTATG
	**Forward**	TGAGAACTGAATTCCATAGG
**hsa-miR-335-3p**	**RT Primer**	GTCGTATCCAGTGCAGGGTCCGAGGTATTCGCACTGGATACGGGTCAGGA
	**Forward**	TTTTTCATTATTGCTCCTGACC
**hsa-miR-342-3p**	**RT Primer**	GTCGTATCCAGTGCAGGGTCCGAGGTATTCGCACTGGATACGACGGGTGC
	**Forward**	TCTCACACAGAAATCGCAC
**hsa-miR-375**	**RT Primer**	GTCGTATCCAGTGCAGGGTCCGAGGTATTCGCACTGGATACGTCACGCGA
	**Forward**	TTTGTTCGTTCGGCTCGC
**hsa-miR-491-3p**	**RT Primer**	GTCGTATCCAGTGCAGGGTCCGAGGTATTCGCACTGGATACGGTAGAAGG
	**Forward**	CTTATGCAAGATTCCCTTCTA
**hsa-miR-644b-3p**	**RT Primer**	GTCGTATCCAGTGCAGGGTCCGAGGTATTCGCACTGGATACGTGTAGGCT
	**Forward**	TTCATTTGCCTCCCTGCC
**hsa-miR-4732-3p**	**RT Primer**	GTCGTATCCAGTGCAGGGTCCGAGGTATTCGCACTGGATACGCAGAACAG
	**Forward**	GCCCTGACCTGTCCTGT

* RT primers were used for cDNA synthesis in each case; Reverse universal and Forward primers were used for real time analysis for each miRNA

### Statistical Analysis

The relative expression was calculated using the equation 2-ΔΔCT where ΔΔCT = Δ CT target miRNA- ΔCT U6. Statistical analysis for qRTPCR was done using unpaired, two way student`s t-test and a probability level of p < 0.05 was considered statistically significant.

## Results

### MicroRNA expression is different in Rectosigmoid area compared to Ascending Colon in UC patients

#### Microarray analysis

**Fig 1:**
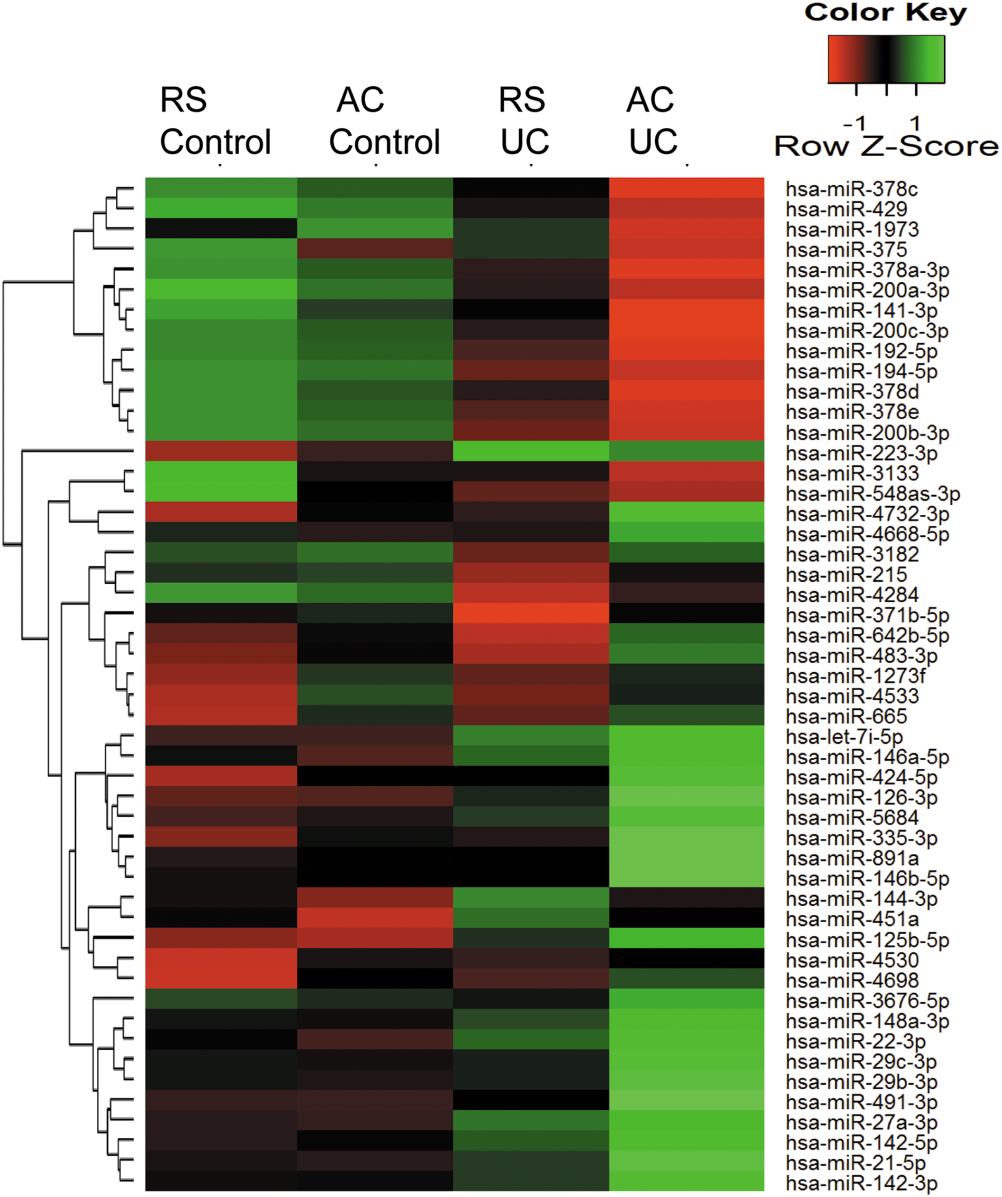
Heatmap and unsupervised hierarchical clustering of miRNAs. Heatmap showing differential miRNA expression in UC patients involving rectosigmoid area (RS) and ascending colon (AC) vs. control. Green color indicates higher than mean intensity and red represents lower than mean intensity.

The site of CRC development in UC is rectosigmoid region in most of the cases. Given the critical role of miRNAs in IBD and its association with CRC incidence, we performed microarray analysis on pinch biopsies from patients where either ascending colon or rectosigmoid area was involved. Fig 1 represents the heatmap for the microarray data describing the expression profile of miRNAs in rectosigmoid area vs. ascending colon of UC and non-IBD controls. We identified 7 miRNA with altered expression in UC patients involving rectosigmoid region (RS) compared to Ascending colon (AC) (GEO Submission- GSE66932). Expression of six miRNAs hsa-miR-146b-5p, hsa-miR-335-3p, hsa-miR-342-3p, hsa-miR-644b-3p, hsa-miR-491-3p, hsa-miR-4732-3p were decreased in patients where RS compared to AC was involved in UC. On the other hand, expression of hsa-miR-141-3p and has-miR-375 was found to be increased in RS compared to AC in UC patients (S1 Fig) Interestingly, hsa-miR-375 did not show any change in expression pattern due to disease condition when compared with the non-IBD subjects however, the changes were seen only between two locations RS and AC. Therefore, we presumed that hsa-miR-375 does not have any impact on the disease condition and thus it was not pursued further. Table 3 represents relative fold changes observed in miRNA expression, deduced from the microarray data of UC patients involving two different sites- RS compared to AC. We observed 0.5 fold decrease in expression of most of the above mentioned miRNAs and only hsa-miR-141-3p exhibited 1.7 fold increase in expression in samples from rectosigmoid area compared to ascending colon during disease condition.

**Table 3:** Fold changes observed in miRNA expression in Rectosigmoid region compared to Ascending colon based on microarray data.

Sr No.	Annotation	Relative fold Change
1	hsa-miR-375	2.17
2	hsa-miR-491-3p	0.49
3	hsa-miR-4732-3p	0.50
4	hsa-miR-141-3p	1.70
5	hsa-miR-146b-3p	0.57
6	hsa-miR-335-3p	0.58
7	hsa-miR-342-3p	0.58
8	hsa-miR-644b-3p	0.59

#### Quantitative Real time PCR analysis of selected miRNAs upregulated or downregulated in microarray analysis

**Fig 2:**
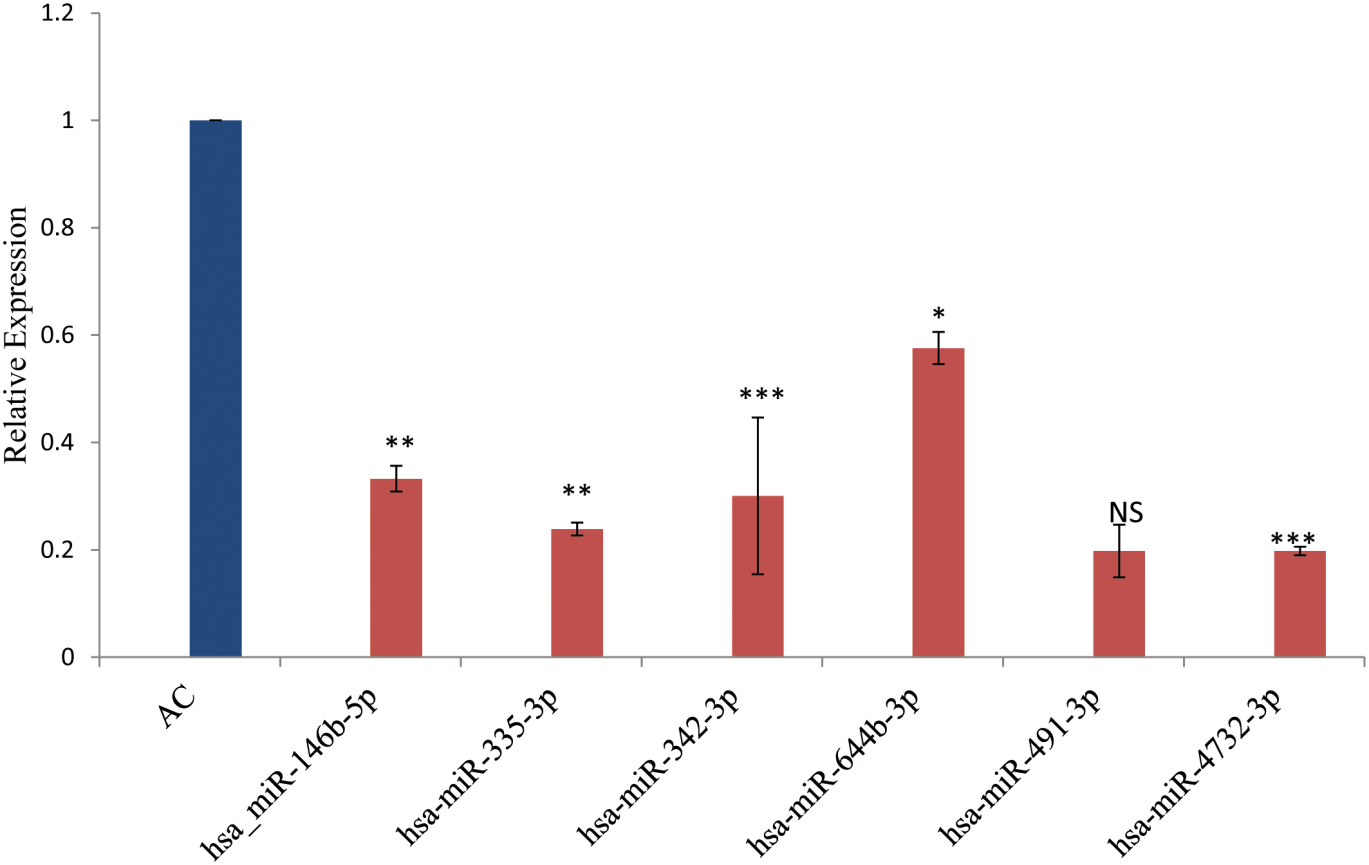
Relative miRNA expression showing downregulated miRNA in AC. Relative miRNA expression by qRT PCR showing downregulated expression of miRNA in UC patients involving rectosigmoid area compared to ascending colon. * represents the level of significance. NS: not significant.

**Fig 3:**
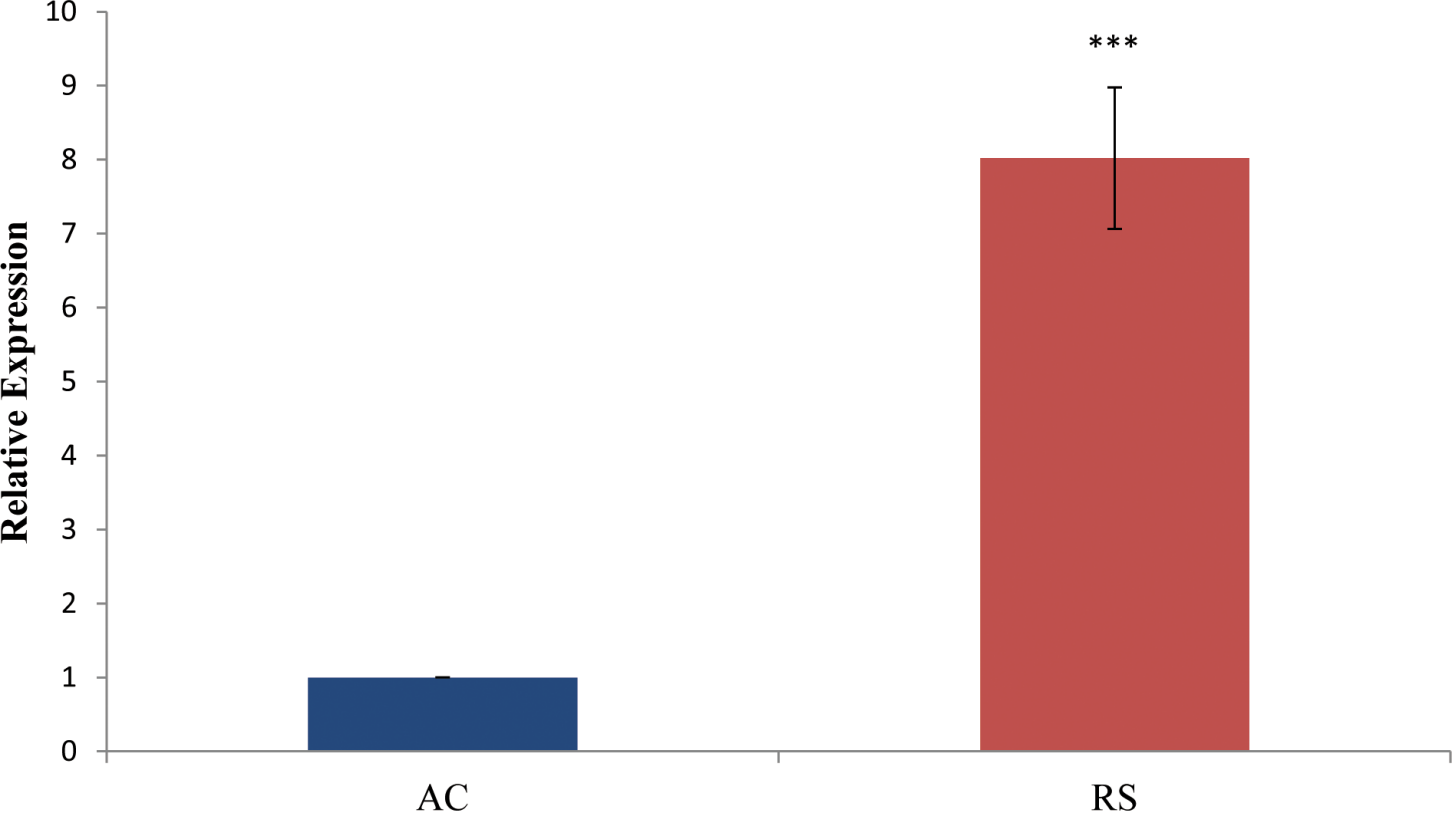
Relative miR-141-3p expression by qRT PCR : miR-141-3p shows eight fold upregulation in rectosigmoid area compared to ascending colon. * represents the level of significance.

Here we focused on miRNAs that were showing region specific differences in UC only. Microarray data was validated by qRTPCR in biopsy samples (20 from ascending colon and 30 from rectosigmoid area) of UC patients and 20 samples from non IBD controls. Expression analysis results using qRTPCR showed a similar pattern as observed in microarray analysis with variation in fold changes (Fig 2). Hsa-miR-146b-5p, hsa-miR-335-3p, hsa-miR-342-3p, hsa-miR-644b-3p and hsa-miR-4732-3p showed significant site specific changes in UC as revealed by microarray data. The expression of hsa-miR-491-3p showed 0.49 fold decrease however it was not significant. The expression of hsa-miR-141-3p exhibited 8 fold increase in expression by real time analysis when samples from rectosigmoid area was compared with the ascending colon (Fig 3).

### Rectosigmoid is the most common site of CRC development

**Fig 4:**
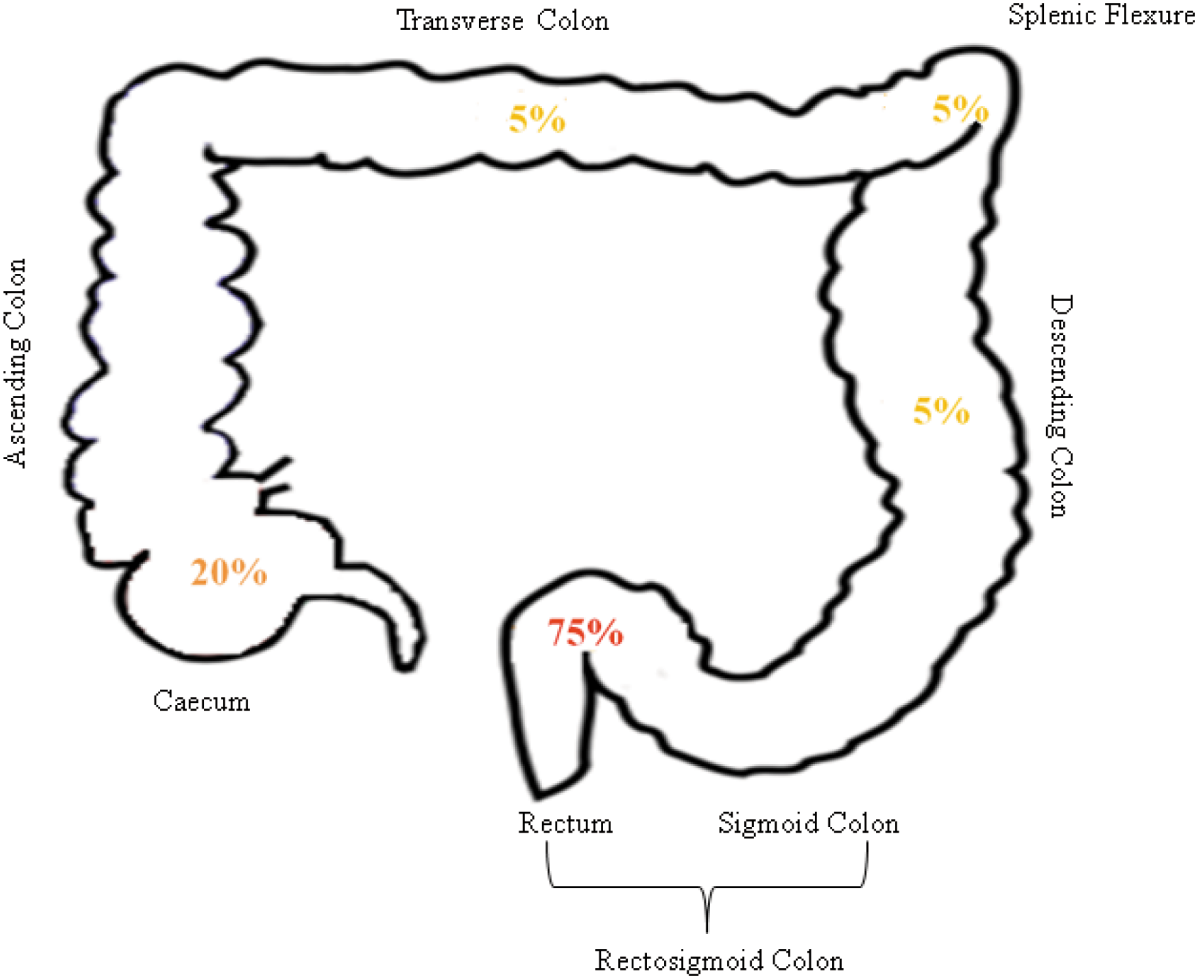
Site specific prevalence of CRC in UC associated cancer patients. 75% of UC cases developed CRC in rectosigmoid area, 10% in caecum, and 5% in decending colon, splenic flexure and transverse colon each.

As per the hospital record, during last 10 years total 20 UC patients were reported to have developed UC associated CRC. We observed that in 15 patients (75%) of the cases, the site of CRC was rectosigmoid area (60% in the rectum and 15% in the sigmoid colon) (Fig 4). There was no case in the hospital based record, where UC-associated CRC developed from ascending colon. There were cases of CRC development in patients where different regions of colon like descending, transverse, caecum and splenic flexure were involved. 14 out of 20 UC cases were male and 7 were females. Extent of disease was either left sided colitis or pancolitis. Mean duration of UC before CRC development was 17.5 years. Pseudopolyps were present only in 26.32% of the UC cases progressing to CRC.

## Discussion

UC patients are considered to be at increased risk of developing CRC. The prevalence of CRC in UC patients in the Asia-Pacific region has been estimated to range from 0.3%-1.8% [15]. Further long-term data to assess the parameters on the cumulative risk attributable to UC and CD are required in the Asia-Pacific region. MiRNAs have been implicated to play an important role in progression of chronic inflammation towards cancer.

Site of CRC in UC patients is reported to be the RS area in most of the UC-associated CRC patients. As per the hospital record of the UC patients, we observed that the site of colorectal cancer in 73.68% of UC associated CRC patients was in RS area of colon where none was recorded from ascending colon region of the affected individual. This supported earlier observations seen in IBD patients turning into CRC [4].

Alteration in miRNA expression has been reported in UC compared to healthy controls. Feng Wu et al, 2008 reported differential expression of 11 miRNAs in UC compared to healthy controls [16]. Ueda et al reported miR-124a as a potential risk marker for UC associated CRC [17]. miRNA were shown to be differentially expressed in mice model for colitis associated tumor [18]. There is no report for differential miRNA expression at different sites of colon of UC patients. We analyzed miRNA expression at RS vs AC of UC patients.

Using microarray analysis and qRTPCR, our results showed that seven miRNA were differentially expressed in active UC patients involving either rectosigmoid area or ascending colon. Our data further confirmed that except hsa-miR-141-3p, six other miRNA were downregulated in patients with rectosigmoid area compared to ascending colon.

Among other miRNA that were downregulated in UC patients involving RS, one important miRNA was miR-146b-5p. MiR-146b has been reported to play important role in linking inflammation and cancer. Tumor suppressive role of this miRNA in carcinogenesis has been confirmed through a study conducted by Kanan et al (2012) while tracking the changes in expression of miRNA in CD patients from non neoplastic tissue to dysplasia and eventually to CRC where the expression miR-146 significantly decreased during transition to CRC [19]. It has an important role in inflammation by regulating NF-kB pathway through IRAK-1 [20]. It has been reported to have inhibitory role in cell proliferation and invasion too [21]. Xiang et al (2014) described miR-146b as a direct target of signal transducer and activator of transcription 3 (STAT3) whose activation is involved in mechanism by which chronic inflammation can contribute to cancer [22]. Tumor suppressive role of miR-146b in breast cancer cells have been proposed where overexpression of miR-146b suppressed NF-κB dependent expression of IL-6 and subsequent invasiveness and mesenchymal phenotype of breast cancer [23].

miR-335 has been reported to play role in metabolic pathway shared by UC and CRC. Inflammatory cytokines serve as important mediators of continuous crosstalk between the immune system and tumor cells and function at multistage of inflammation- associated tumor process. MiR-335 is known to mediate crosstalk between cytokine-cytokine receptor interaction and chemokine signaling pathway through the regulation of chemokine families [24]. It acts as tumor suppressor by regulating the expression of ZEB in CRC. Patients with reduced miR-335 had a poor rate of overall survival. In vitro studies showed that by enhancing the expression of miR-335, CRC cell migration and invasion can be inhibited [25]. Stage dependent differential expression of miR-335 with clinical progression of CRC and as a prognostic marker for metastasis in CRC has also been reported [26]. Decreased expression of this miRNA observed in our study corroborates the hypothesis that the patients of UC involving rectosigmoid area are prone to develop CRC in future.

Decreased expression of miR-342-3p observed by us in UC patients involving rectosigmoid area supports the study carried out in an integrated miRNA and mRNA expression profiling of inflammatory breast cancer sub type [27]. MiR-342 has also been shown to act as a tumor suppressor gene in CRC development by regulating aberrant DNA hypermethylation [28].

High levels of miR-644b-3p were found to be associated with shorter overall survival of acute myeloid leukemia (AML) [29]. However, so far no association studies have been reported in context to any inflammatory disease. Similarly, decreased expression of miR-4732-3p observed in this study, has not been reported so far in the literature.

Increased expression of miR-141-3p observed in microarray data was validated by qRTPCR in biopsy samples of individual UC patients with RS was compared with AC. We found eight fold increased expression of only one miRNA, miR-141-3p in tumor prone RS area of colon. This context dependent expression pattern observed in the present study supported earlier study carried out in tumour samples of stage III CRC patients where increased expression was observed [30]. Cheng et al. also reported increased expression of miR-141 and correlated with different stages of CRC. They also predicted circulating miR-141 as potential biomarker for metastatic colon cancer [31]. MiR-141-3p was reported to promote proliferation of non-small cell lung cancer cells by PH domain leucine-rich-repeats protein phosphatase 1 (PHLPP1) and PHLPP2 [32].

MiRNA are reported as an important link between inflammatory diseases and cancer [2]. In this study we found dysregulation of seven miRNAs. Out of seven, four miRNAs are reported earlier to have role in different type of cancers. Three dysregulated miRNAs, miR-146b-5p, miR-335, miR-342, were reported as tumor suppressors in different studies. These three miRNAs are reported to play important role in inflammation associated cancers and their overexpression decreased the proliferation rate of cancer cell lines [19, 21, 24–25, 27]. We observed decreased expression of these miRNAs in tumor prone RS area of colon compared to AC of UC patients. Overexpression of miR-141-3p was reported to increase the proliferation in non-small cell lung cancer [32]. We found increased expression of miR-141-3p in tumor prone RS area of colon.

Based on our data and the important role of dysregulated miRNAs in inflammation and the development of cancer, we propose that site -specific change in miRNA expression may be one of the contributing factor for increased incidence of UC associated CRC at RS region of colon.

## Conclusion

**Fig 5:**
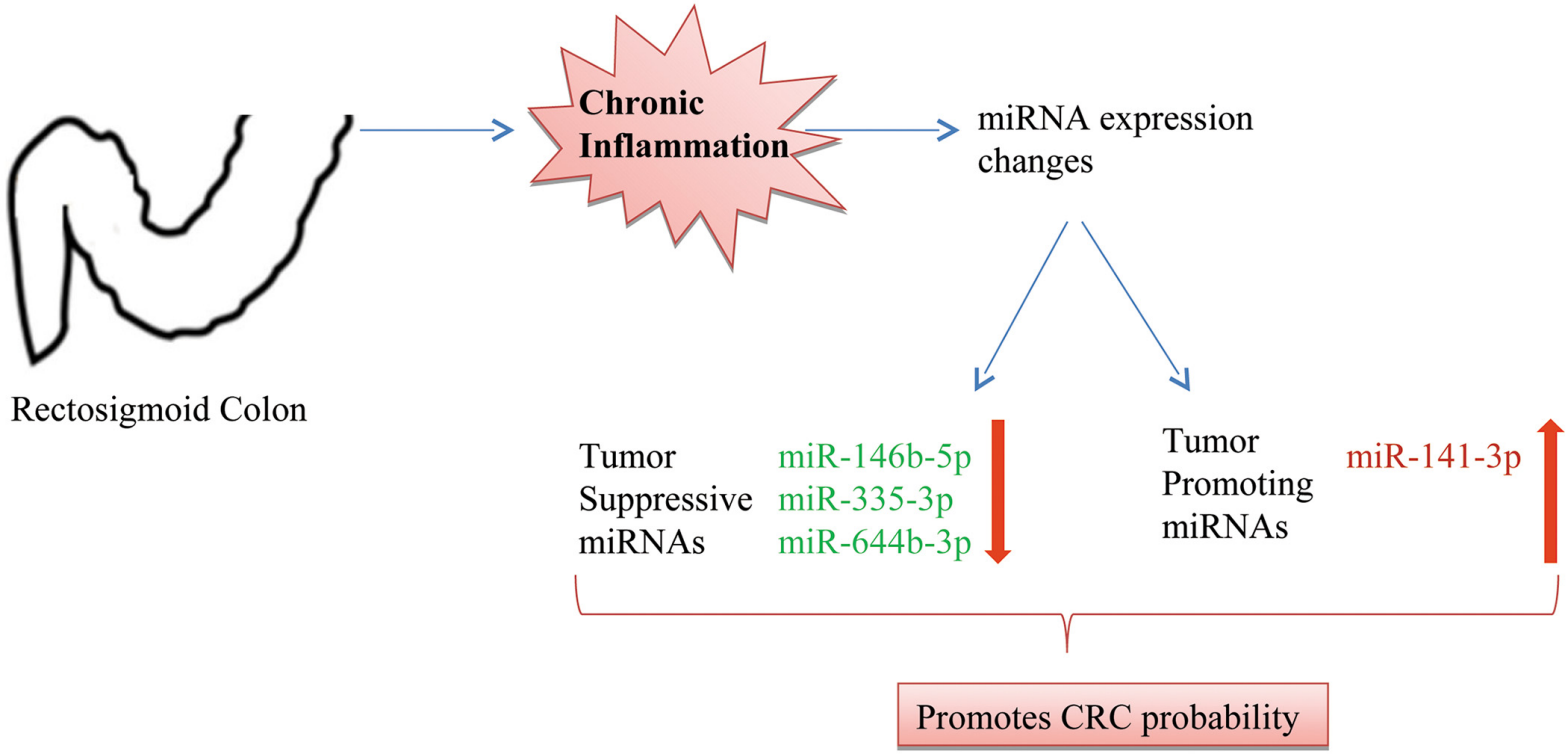
Increased probability of CRC development in Rectosigmoid region of CRC patients. Chronic inflammation in colon of UC patients causes site specific miRNA expression changes. Decrease in expression of tumor suppressive miRNAs (miR-146b-5p, miR-335-3p, miR-644b-3p) and increase in expression of tumor promoting miRNA (miR-141-3p) were observed. The expression changes may lead to increased probability of CRC initiating in rectosigmoid region of UC patients.

Our study indicated that site specific miRNA expression may be one of the contributors in CRC development in rectosigmoid colon in UC patients. Based on our analysis we propose that increase in expression of tumor promoting miRNA and decrease in expression of tumor suppressive miRNA may be one of the reasons for increased occurrence of CRC in the rectosigmoid area during ulcerative colitis (Fig 5). Functional studies are warranted to validate our data. This information will help to distinguish the UC subtypes based on miRNA profiling to assess disease activity and suggest appropriate interventions before the disease progresses to CRC.

## Supporting Information

S1 Fig miRNA expression by Microarray analysis. Relative miRNA expression in Rectosigmoid region compared to Ascending colon of UC patients by microarray analysis.
(TIF)
