# Supplementary figures and images for: Site-Specific MicroRNA Expression May Lead to Different Subtypes in Ulcerative Colitis
Source: PLoS One. 2015 Nov 16;10(11):e0142869. doi: 10.1371/journal.pone.0142869 (PMC4646509; doi:10.1371/journal.pone.0142869)

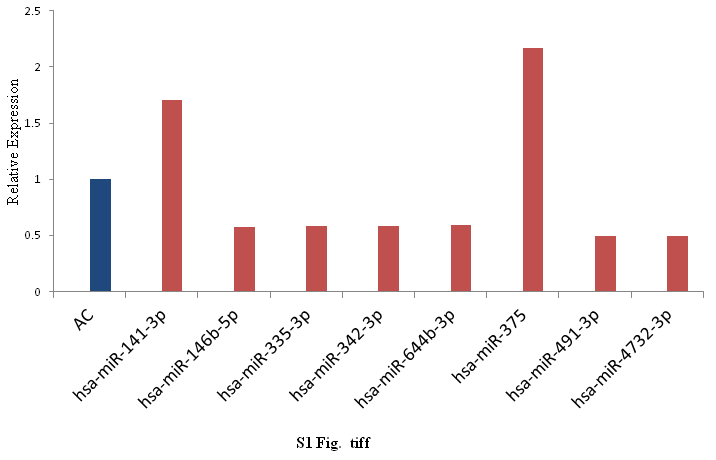

Supplement: S1 Fig — Relative miRNA expression in Rectosigmoid region compared to Ascending colon of UC patients by microarray analysis. (TIF) [file pone.0142869.s001.tif]
